# Opioid prescriptions following behavioral health training among primary care providers

**DOI:** 10.1186/s12909-024-06289-y

**Published:** 2024-11-26

**Authors:** Shutong Huo, Tim A. Bruckner, Abhery Das, Glen L. Xiong, David Marcovitz, Ariel B. Neikrug, Robert McCarron

**Affiliations:** 1 Department of Health, Society, and Behavior, UC Irvine Joe C. Wen School of Population & Public Health, UCI Health Sciences Complex, 856 Health Sciences Quad, Irvine, CA 92697 − 3957 USA; 2 University of California Irvine, Center for Population, Inequality, and Policy, Irvine, CA USA; 3 Heath Policy & Administration, School of Public Health, University of Illinois Chicago, Chicago, IL USA; 4 University of California, Davis, Psychiatry and Behavioral Sciences, Sacramento, CA USA; 5 Department of Psychiatry and Behavioral Sciences, Vanderbilt University Medical Center, Nashville, TN USA; 6 Department of Psychiatry and Human Behavior, University of California Irvine School of Medicine, Irvine, CA USA

**Keywords:** Opioid prescribing behavior, Psychiatric Training, Primary care providers, Substance Use disorders, Opioid Crisis

## Abstract

**Background:**

Overdose deaths due to opioids are a major concern in the United States. Physicians often report inadequate training in chronic pain and substance use disorder management. Here, we evaluate whether a specialized program, the Train New Trainers Primary Care Psychiatry (TNT PCP) Fellowship, affected opioid prescription practices among primary care physicians.

**Methods:**

We retrieved information from a publicly insured health program in Southern California on 11,975 patients and 180 primary care providers (PCPs) engaged in care between 2017 and 2021. Of the 180 PCPs, 38 received TNT training and 142 did not. We considered a patient as exposed to the provider’s TNT “treatment” if they received care from a provider after the provider completed the 1-year fellowship. We utilized the number of opioid prescriptions per patient per quarter-year as the key independent variable. Linear regression models controlled for provider characteristics and time trends. Robustness checks included clustering patients by provider identification.

**Results:**

Post-TNT training, PCPs prescribed fewer than expected opioids. This result remains robust after controlling for several covariates (coef: − 0.209 ; standard error = 0.052, *p* < 0.001) as well as after clustering patient observations by provider.

**Conclusion:**

In a large Southern California healthcare system, the TNT training program preceded a reduction in primary care providers’ prescription rates of opioids. If replicated in larger samples, a low-cost provider training program has the potential to promote more judicious use of opioids for pain management. We encourage more studies to understand the program’s long-term impact on physician behavior and, potentially, on patient outcomes.

## Introduction

In the United States, overdose deaths, primarily involving opioids, remain a leading cause of accidental and injury-related death [1, 2]. The opioid crisis in the United States, driven in part by prescription opioids, is a significant public health concern [3]. Deaths resulting from prescription opioid overdoses experienced a sharp escalation from the late 1990s until 2017 [4]. In 2022, prescription opioid overdose death still count for about 13.6% of overdose death involving any opioid, continuing to contribute to the opioid crisis in the US [4]. Moreover, in California, over 90% of opioid overdose deaths involve prescription opioids [5]. Earlier research finds that escalating prescription opioid usage has contributed to a surge in overdose rates and mortality [6, 7]. Also, **increasing the dose of prescribed opioids is positively associated with a higher risk of overdose** [8–10].

Patients with untreated psychiatric disorders and those on psychotropic medications are at higher risk for opioid misuse and dependence, with mental health symptoms leading to increased self-reported pain limitations and greater opioid use [11–13]. Additionally, psychiatric symptoms, particularly depression and anxiety, experience more adverse opioid outcomes, such as misuse and poor treatment responses [14]. Despite this need, more than 30% of patients seeking primary care do not have access to specialty psychiatric services [15]. Compounding this issue, many physicians report feeling inadequately trained to address chronic pain and substance use disorders [16–18]. These gaps in medical education highlight the urgent need for enhanced training to equip clinicians with the skills necessary to address the complex interplay between psychiatric disorders, chronic pain, and opioid prescribing.

Continuing medical education (CME) plays a pivotal role in refining provider competency, specifically in opioid prescribing and chronic pain management. Programs such as the SCOPE of Pain and others have been identified as effective in enhancing both provider confidence and their adherence to CDC recommended practices, given they dedicate substantial hours to these specific educational pursuits [19, 20]. Furthermore, these programs are instrumental in fostering improved knowledge, attitudes, and changes in clinical practices toward safer opioid prescribing. A study by Moride further substantiates these findings, underlining the positive impact of continuing medical education on chronic pain management outcomes [21]. Thus, the incorporation of continued medical education remains a critical strategy in ensuring appropriate, patient-centered opioid prescribing practices.

Evaluation of many such CME programs remains limited in that the downstream evaluation on patient outcomes resulting from that education lack a comparison group treated by providers unexposed to the education intervention. This type of design may lead to confounding by external factors unrelated to the education programs. Nevertheless, previous evaluations suggest an improvement in knowledge and attitudes towards safe opioid prescribing [21]. Most of these programs primarily target opioid prescribing and pain management. **However**,** earlier research found that while opioids account for the majority of single-medication deaths**,** nearly 40% of unintentional prescription-related deaths involve a combination of prescription drugs**,** illicit substances**,** alcohol**,** and over-the-counter medications** [22]. **Therefore**,** for effectively managing psychiatric comorbidity and promoting judicious opioid prescribing**,** an integrated medical education program like the TNT PCP Fellowship could prove useful. Such a program could address not only substance use but also a wider range of mental disorders.**

We developed a CME program to deliver psychiatric training for primary care providers (PCPs). The Train New Trainers Primary Care Psychiatry (TNT PCP) Fellowship, implemented by the University of California, Irvine (UCI), is a 12-month longitudinal training initiative designed to improve the psychiatric expertise of PCPs. The program aims to enhance their proficiency in the identification, prevention, and management of psychiatric disorders frequently encountered in primary care settings. A prior assessment of the program revealed that participants exhibited significant improvements in both their psychiatric clinical knowledge and their attitudes toward mental health stigma after they finished the training [23]. In addition, the study also found that the prescription rates of antidepressants among patients treated by TNT PCP increased after training compared with non-TNT PCP patients, which indicates the improvement in behavioral health services among TNT PCP [24].

Our **study** investigates whether exposure to a TNT PCP fellowship reduces the likelihood of patients receiving opioid prescriptions. In this study, we examine data from approximately 12,000 patients enrolled in a publicly funded health program in Southern California, who are connected to **180** PCPs. The findings from this study are intended to offer preliminary insights into the potential benefits of broader implementation of the TNT program across California, to enhance the appropriateness of opioid prescribing practices within primary care settings.

## Methods

### Program description

The TNT PCP Fellowship offers a structured educational program, delivering over 50 h of training outside of the clinic setting throughout a calendar year. A primary component of this training includes two large-group conferences that combine interactive learning and small-group discussions designed to optimize participants’ ability to apply the new information to the clinic setting and patient interactions. Throughout the remainder of the year, participants engage virtually with TNT faculty via bi-weekly sessions and monthly mentorship meetings. TNT faculties hold expertise in primary care specialties, such as internal medicine, pediatrics, or family medicine, as well as psychiatry, with most possessing dual training in both fields. The curriculum covers a broad spectrum of behavioral health topics and skills in the primary care setting, including psychiatric interviewing, mental status examinations, and the management of psychiatric conditions such as mood, anxiety, sleep, **psychosis**, substance use, somatic symptoms, and personality disorders [23]. **TNT training included chronic pain management**,** with participants spending an estimated 2–4 h on this topic**,** with additional discussion opportunities in mentorship-based sessions. The curriculum also addresses opioid use disorder (OUD) and other substance use disorders**,** providing PCPs with integrated approaches to managing these complex conditions.** Key elements also include suicide risk assessment, pain management within psychiatry, fundamentals of psychopharmacology, cultural formulation, motivational interviewing, and cognitive behavioral therapy. The training underscores the significance of empathy and shared humanity as foundational principles for delivering effective and compassionate patient care. Furthermore, TNT offers alumni of the fellowship continued professional development opportunities, including free relevant training and mentorship.

### Variables and data

We retrieved PCP and patient data from Inland Empire Health Plan (IEHP), a managed care organization that ranks among the top ten largest health plans within California’s Medicaid program (Medi-Cal). IEHP’s network comprises over 8,000 healthcare providers and currently serves more than 1.6 million residents in Riverside and San Bernardino counties. The patient population is representative of the diverse population of Californians racially and ethnically, with eligibility for Medi-Cal determined by means-tested low-income. **The IEHP patient population is racially and ethnically diverse**,** with Hispanic individuals comprising 47.4% of the sample.**

We identified 40 PCPs within the IEHP network who participated in the TNT program and completed their training in different years (10 in 2018, 13 in 2019, and 17 in 2020). Additionally, we obtained a control group comprising 152 PCPs who did not attend TNT training but practiced in similar clinical environments as their trained counterparts. Out of the 192 providers included in our analysis, 180 were documented as having prescribed opioids during the study period. We then extracted the medication prescription histories of 11,975 patients who had visits associated with these **180** PCPs from IEHP’s clinical records. The patient records included both the date of each visit and the prescribed medications. **We also obtained sociodemographic data for a subset of these patients for descriptive purposes**. Consent was waived by the University of California, Irvine Institutional Review Board (protocol # 20216483).

The dataset covers the time frame from one year before the initiation of TNT training to one year following the completion of the most recent cohort’s training, allowing for a comprehensive evaluation of opioid prescribing patterns pre- and post-training. Over the five years, we identified a total of 49,926 opioid prescriptions within the IEHP dataset. The primary outcome measure in our study was the number of opioid prescriptions per patient per quarter. We calculated this by aggregating the total number of opioid prescriptions from each encounter and summarizing this data on a quarterly basis.

### Analysis

We first compared the quarterly opioid prescription rates among patients treated by TNT-trained PCPs before and after the providers completed TNT training. To define exposure, we classified a “TNT-exposed period” as the timeframe during which a patient received care from a PCP who had already completed the TNT program. An “unexposed period” referred to instances where a patient was treated by a PCP prior to their TNT training. Recognizing the potential for secular trends in prescription rates—trends that could coincide with but not be directly caused by the TNT training, particularly in the latter half of the study period 2017–2021 —we implemented several strategies to reduce this bias. We applied ordinary least-squares regression techniques and incorporated a time-trend variable, continuous quarter-year, as a control within our regression. For PCPs who underwent TNT training, the quarter during which training began was excluded from the analysis, leaving a uniform 19-quarter observation period for each patient. For patients under the care of non-TNT-trained PCPs, we randomly excluded one quarter to maintain consistency in the length of the observation period.

Additionally, we improved the estimation of our regression by including patients who were treated by PCPs in IEPH but never participated in the TNT program. These patients contributed to person-time throughout the study period, aiding in the control of general trends in prescription rates. **To further account for potential confounding factors related to PCP characteristics**,** such as prescribing behavior and the likelihood of participating in TNT training**,** we included variables representing the PCP’s gender**,** specialty**,** and age in our regression** [25–27]. **We also controlled for seasonality by including four seasons as categorical variables in the models**,** accounting for potential variations in opioid prescribing patterns across different year seasons** [28].

Our regression analysis was structured with an indicator variable for whether a prescription was issued after the PCP completed TNT training (value = 1) or not (value = 0). Similarly, another we specified an indicator variable of whether a PCP had ever attended the TNT training (value = 1) or not (value = 0**). The interaction term between the time variable and TNT training served as our primary independent variable**,** capturing the effect of TNT training on prescription behavior over time. This interaction allowed us to determine whether the changes in prescribing patterns were specifically due to TNT training**,** independent of any overall trends in prescribing behavior across all PCPs over the study period.** We calculated all coefficients and standard errors both with and without clustering patient observations by PCP identification. Data analysis was conducted using STATA 16.

## Results

**Fig. 1:**
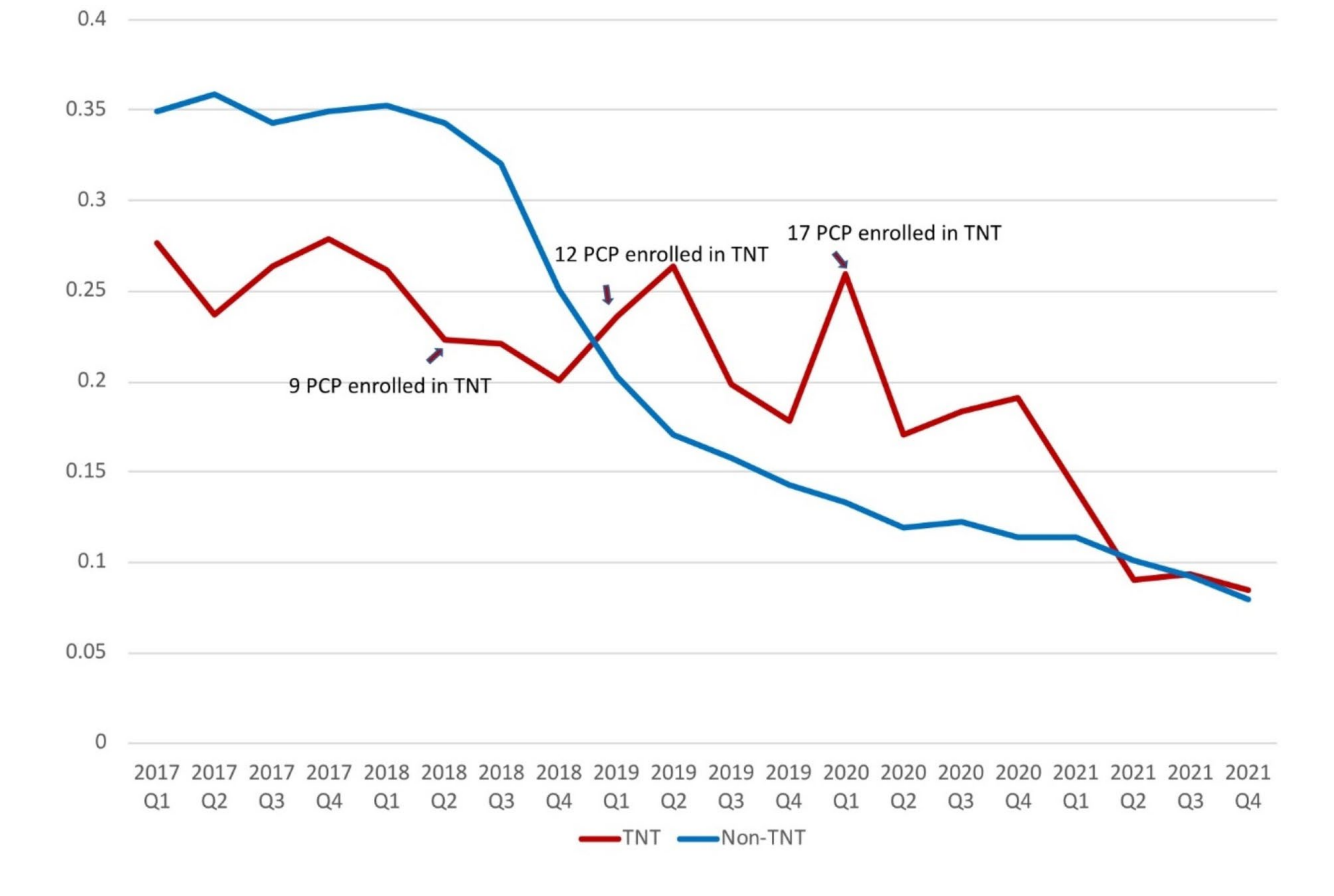
Mean prescriptions per patient per quarter for opioids by TNT providers and non-TNT providers from 2017 to 2021. Arrows indicate timing of training and number of TNT providers trained in that cohort

Table 1 shows sociodemographic characteristics of a subset of the patient population. Non-Hispanic white and Hispanic patients comprise over 70% of the sample. **Over half of the sample has a “high” adjusted clinical grouping score**,** which indicates a high risk for severe morbidity and/or prescription opioid misuse and dependence. In addition**,** prior to TNT training**,** TNT PCPs averaged approximately 0.250 opioid prescriptions per quarter. Following the training**,** this average decreased to 0.141 prescriptions per quarter.** Figure 1 shows average quarterly opioid prescriptions per patient by TNT and non-TNT provider status. **We observe broader downward trends in opioid prescribing in both groups with short-term fluctuations.** The figure also indicates the timing, and size, of the participation of TNT providers from three cohorts (2018, 2019, 2020) in the training.

**Table 1:** Characteristics of **a subset of patients (6**,**578)** seen by 38 TNT-trained providers (before and after training) as well as by 142 non-TNT trained providers

	Patients seen by TNT providers		Patients seen by non-TNT providers	
	N	%	N	%
Age				
0–20	4	0.2	11	0.24
21–40	445	22.5	955	20.76
41–60	875	44.24	2143	46.59
61–80	602	30.43	1399	30.41
81+	45	2.28	92	2
Homeless	75	3.79	230	5
Ethnicity				
Non-Hispanic white	730	36.91	1706	37.09
Non-Hispanic Black	283	14.31	688	14.96
Hispanic	718	36.3	1696	36.87
Asian	35	1.77	65	1.41
Others	205	10.36	445	9.67
Adjusted Clinical Group Risk level				
Low	544	27.5	1288	28.0
Moderate	390	19.72	1033	22.46
High	1037	52.43	2279	49.54

Table 2 presents regression results estimating the relation between TNT training and quarterly opioid prescriptions per patient. The coefficient of interest is the interaction term of TNT provider and the time-period *after* TNT training. The left column of the table shows the Model 1 as the base model; compared to expected levels, the patients of TNT providers, *after* the TNT training, receive on average 0.226 fewer opioid prescriptions per quarter following the TNT training (*p* < 0.0001). Model 2 controls for provider characteristics, seasonality, and time trends, indicating that PCP age, gender, and specialty show associations with the quarterly opioid prescriptions per patient. Adjusting for provider characteristics in Model 2 does not substantially affect the primary results of TNT training (coef: -0.226 fewer prescriptions per quarter on average [*p* < 0.001]).

**Table 2:** Coefficients estimating the rate of prescriptions of opioid per quarter among patients in the Inland Empire Health Program (*n* = 11,975) as a function of provider characteristics, time, whether the provider ever received the “Train the Trainer” program, and the interaction of time and provider receipt of the TNT program

Variables	Rate of prescriptions		
	Model 1	Model 2	Model 3
Time	0.117** (0.044)	0.264*** (0.043)	0.264*** (0.029)
TNT training	0.038*** (0.005)	0.007 (0.005)	0.007 (0.049)
Time*TNT training	-0.226*** (0.044)	-0.209*** (0.041)	-0.209*** (0.052)
Male provider (referent: Female)		0.017*** (0.004)	0.017 (0.034)
Specialty			
Family Practice (Omitted)			
General Practice		0.082*** (0.009)	0.086 (0.069)
Internal Medicine		-0.001 (0.006)	-0.001 (0.042)
Nurse		-0.040*** (0.007)	-0.040 (0.035)
Physician Assistant		-0.158*** (0.013)	-0.158** (0.049)
Age			
30–39 (Omitted)			
40–49		-0.004 (0.005)	-0.004 (0.025)
50–59		-0.012 (0.007)	-0.012 (0.030)
60–69		0.118*** (0.005)	0.118* (0.054)
70+		0.008*** (0.013)	-0.161* (0.074)
Quarterly trend		-0.016*** (0.000)	-0.016*** (0.004)
Seasonality			
Jan.-Mar.(Omitted)			
Apr.-Jun.		0.002 (0.005)	0.002 (0.007)
Jul.-Sept.		0.006 (0.005)	0.006 (0.007)
Oct.-Dec.		0.008 (0.005)	0.008 (0.008)
Constant	0.212*** (0.002)	0.329*** (0.006)	0.039*** (0.048)

*p < 0.05; **p < 0.01; ***p < 0.001

Notes: Model 1 (left column) represents the base model;

Model 2 controls for provider characteristics, seasonality, and time trends

Both Models 1 and 2 treat all patient observations as statistically independent

Model 3 was clustered by providers using the “cluster” standard error estimation routine in STATA

We then adjusted for potential provider-level clustering, an approach justified if providers demonstrate consistent prescribing behaviors across their patients, thereby leading to correlated patient experiences within each provider’s practice. We utilized the “cluster id” option in STATA, which considerably reduced the effective sample size from 11,975 prescriptions to 177 providers. As anticipated, clustering diminished the precision of standard error estimates for the interaction term between TNT provider status and the post-training period (Model 3, standard error [SE] = 0.046 vs. Model 2 SE of 0.044). The point estimate, however, remained consistent and statistically detectable (coef: 0.226; *p* < 0.001).

## Discussion

Behavioral health and substance use training for primary care providers remains an essential factor in curbing the opioid epidemic in the US. Rigorous evaluations of training programs in this area to date, however, remain limited. Here we examined the relation between a novel Train-the-Trainer (TNT) program and opioid prescribing behavior among **180** physicians, serving **11**,**975** patients, over a three-year period. Importantly, we include both a TNT-trained “treatment group” and a control group of physicians who never received the provider training. Our study findings point to a decline in the average quarterly opioid prescriptions per patient after the TNT training. **When scaled to all patients in our study**,** this results in approximately 2**,**706 fewer opioid prescriptions per quarter and a total annual reduction of over 10**,**800 prescriptions. Such a decrease may have population-level implications**,** as fewer prescriptions can help mitigate the risks of opioid misuse**,** dependence**,** and overdose. By reducing the overall number of opioid prescriptions**,** this intervention highlights the potential of education programs to drive meaningful changes in prescribing behaviors**,** contributing to improved patient safety and public health outcomes.**

While we observe a general downward trend in opioid prescriptions in both PCP groups, the TNT-trained providers showed a more pronounced decrease compared to their non-TNT counterparts. This decrease could suggest a positive impact of TNT training in facilitating more judicious opioid prescribing habits. A prior assessment of TNT revealed that upon completion of their training, PCP trainees exhibited marked improvements in their attitudes towards mental health stigma as well as their clinical understanding of psychiatry [23]. Furthermore, our evaluation of the program indicates a higher prescription of antidepressants among patients treated by TNT providers, suggesting a more appropriate treatment approach towards depressive symptoms [24]. Notably, literature indicates that patients with untreated psychiatric disorders are more likely to use significantly more opioids compared to those without such disorders [11]. Consequently, addressing psychiatric issues more effectively could be key in reducing reliance on opioids. Also, gains in empathic communication skills among our PCPs could have downstream effects on engaging patients successfully without resorting to opioid prescribing.

As the prevalence of opioid use disorder increases alongside opioid overdose deaths, implementation of Prescription Drug Monitoring Programs (PDMPs), policies increasing access to substance use care, and insurance mandates attempt to mitigate extensive opioid prescription use [15]. Supply-side policies, such as PDMPs or regulation of pain clinics, show a reduction in opioid prescriptions, whereas other scholars find no relation or an increase in opioid overdoses [15]. Literature on patients with Medicaid across the US reports a 44% decline in opioid prescriptions used to treat pain from 2016 to 2019 [29]. This decline coheres with our findings in that opioid prescriptions among Inland Empire Medicaid enrollees decline after behavioral health training for primary care providers. Targeted training for those issuing opioids to low-income patients may also drive a decline in the broader US. Given that patients with Medicaid have a greater prevalence of opioid use disorder and opioid prescriptions than individuals with other types of insurance, further research would benefit from understanding specific changes in provider behavior in this high-risk population [29]. Standardized assessments for treating pain, such as the implementation of opioid prescribing guidelines from the Centers for Disease Control and Prevention (CDC), may particularly benefit providers treating a higher volume of low-income patients [30].

Limitations of our study include the relatively few TNT-trained PCPs included in our regional analysis. We also do not have detailed sociodemographic and clinical outcome data on all IEHP patients, which limited our ability to examine subgroup differences and clinically relevant outcomes other than opioid prescriptions. **Also**,** we acknowledge the lack of detailed data for the control group of 152 PCPs**,** including their patient load and other practice information.** Furthermore, it is likely that patients with a high adjusted clinical grouping score—that is, those with several comorbidities—may seek care from a team of PCPs. To the extent that prescribing behaviors among a team of providers may more accurately gauge the totality of opioid prescriptions, future work focusing on provider teams may be warranted. **Additionally**,** while our study suggests a relationship between improved psychiatric care and reduced opioid prescribing**,** further research should confirm this finding and assess the extent to which new prescribing levels represent appropriate treatment (rather than**,** for instance**,** under-treatment). Such work could incorporate diagnosis information and track individual prescription changes.** Nonetheless, we believe that temporal changes in enrollment volume do not bias our findings, as we meticulously account for time trends in prescription rates. Lastly, the decision of PCPs to participate in TNT training is not random. Those who opt-in may display a stronger motivation to treat individuals with psychiatric disorders. However, we deem the non-random selection into the TNT training unlikely to skew results, **as both unadjusted analyses of within-PCP prescription rates pre- and post-TNT training** (involving only TNT-trained providers) and adjusted regression analyses with a broader set of PCP controls yield comparable outcomes.

Our future work plans to extend the reach of TNT-trained PCP data beyond Riverside and San Bernadino counties to cover a larger region in California. Although we currently have no basis to suspect unique prescribing behaviors in these counties, larger-scale studies are necessary to affirm their representativeness. In addition, larger studies confirming our results have the potential to profoundly impact clinical practice and medical education, including post-graduate studies and continuing professional development. The UCI TNT PCP Fellowship is designed and implemented by dual-boarded clinicians in primary care psychiatry taking into account the data regarding components of successful programs, including continued educational interactions, a continuous relationship between teacher and learner, interactive participation, and clinical relevance [31, 32]. The fellowship emphasizes training PCPs in the prevention, assessment, and administration of psychiatric care within a clinical setting while considering the multiple demands on PCPs. A broader set of PCPs could benefit from scaled-up TNT PCP training, potentially helping to close the significant psychiatric treatment gap that is especially prominent among publicly-insured patients. **This scale-up**,** while not designed to curb opioid prescriptions**per se, **may hold potential for these positive “spillovers” to the management of substance use disorders. Furthermore**,** studies suggest that reductions in opioid prescribing may disproportionately affect minority groups**,** particularly Black and Hispanic individuals**,** who already receive opioids at lower rates and dosages compared to White patients** [33, 34]. **Given the high proportion of Hispanic patients in our study population and the existing data on inequities in opioid prescribing**,** future work should investigate whether reductions in opioid prescribing disproportionately affect minority groups**,** particularly Hispanic individuals.**

We infer from these results that the psychiatric training among TNT-trained providers may have led to more comprehensive mental health assessments and appropriate psychiatric disorders treatment, which resulted in more cautious and discerning opioid prescribing practices. If findings are replicated in larger studies, this low-cost intervention holds great potential to reduce what many perceive to be over-prescribing of opioids among PCPs for pain management. Once future data become available, we aim to explore the long-term effects of TNT training on PCP prescribing behavior, patient outcomes, and healthcare utilization. Our plan includes monitoring TNT-trained providers’ prescribing habits over several years to see if they maintain the initial reduction in opioid prescriptions. This evaluation can reveal if the training impact lasts or fades over time. A significant metric of any intervention’s efficacy lies in its effect on patient outcomes. Thus, in the context of TNT training, we plan to track patient factors like pain scores, functionality, quality of life, rates of opioid misuse, and overdose incidents over an extended period. These observations will enable us to determine whether changes in prescribing behaviors lead to improved patient health outcomes.

## Conclusion

This study indicates a relation between the TNT program and reduced opioid prescription patterns among primary care providers. Enhanced psychiatric training, as provided by the TNT program, may cultivate more cautious and discerning opioid prescribing practices. Future work will monitor long-term effects and explore the program’s influence on other crucial health and healthcare outcomes.

## Data Availability

The data that support the findings of this study are available from Inland Empire Health Plan (IEHP) but restrictions apply to the availability of these data, which were used under license for the current study, and so are not publicly available.
